# The ribosome stabilizes partially folded intermediates of a nascent multi-domain protein

**DOI:** 10.1038/s41557-022-01004-0

**Published:** 2022-08-04

**Authors:** Sammy H. S. Chan, Tomasz Włodarski, Julian O. Streit, Anaïs M. E. Cassaignau, Lauren F. Woodburn, Minkoo Ahn, Georg Johannes Freiherr von Sass, Christopher A. Waudby, Nediljko Budisa, Lisa D. Cabrita, John Christodoulou

**Affiliations:** 1 Institute of Structural & Molecular Biology, Department of Structural & Molecular Biology, University College London, London, UK and Department of Biological Sciences, Birkbeck College, London, UK; 2 Institute of Chemistry, Technische Universität Berlin, Berlin, Germany; 3 Faculty of Science, University of Manitoba, Winnipeg, Manitoba Canada

**Keywords:** Protein folding, Solution-state NMR, Molecular modelling, Cryoelectron microscopy

## Abstract

Co-translational folding is crucial to ensure the production of biologically active proteins. The ribosome can alter the folding pathways of nascent polypeptide chains, yet a structural understanding remains largely inaccessible experimentally. We have developed site-specific labelling of nascent chains to detect and measure, using ^19^F nuclear magnetic resonance (NMR) spectroscopy, multiple states accessed by an immunoglobulin-like domain within a tandem repeat protein during biosynthesis. By examining ribosomes arrested at different stages during translation of this common structural motif, we observe highly broadened NMR resonances attributable to two previously unidentified intermediates, which are stably populated across a wide folding transition. Using molecular dynamics simulations and corroborated by cryo-electron microscopy, we obtain models of these partially folded states, enabling experimental verification of a ribosome-binding site that contributes to their high stabilities. We thus demonstrate a mechanism by which the ribosome could thermodynamically regulate folding and other co-translational processes.

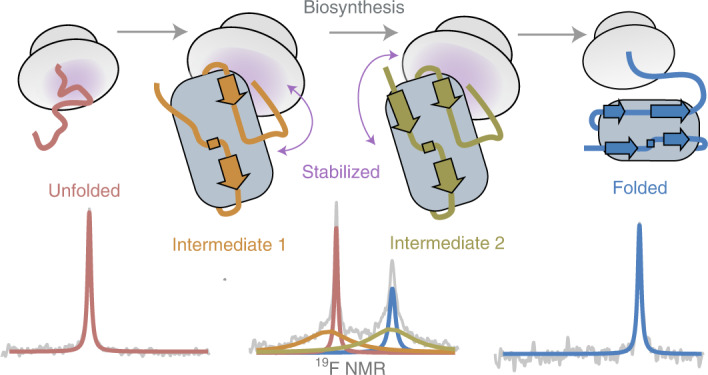

## Abstract (web summary)

Most proteins must fold co-translationally on the ribosome to adopt biologically active conformations, yet structural, mechanistic descriptions are lacking. Using ^19^F NMR spectroscopy to study a nascent multi-domain protein has now enabled the identification of two co-translational folding intermediates that are significantly more stable than intermediates formed off the ribosome, suggesting that the ribosome may thermodynamically regulate folding.

## Main

For most proteins, folding occurs concurrently with translation on the ribosome^1,2^, providing an essential means to avoid the accumulation of misfolded and aggregated states implicated in many human diseases^3^. Analogous to the molecular chaperones that it recruits^4,5^, the ribosome itself is increasingly thought to directly assist the folding process^2^. From the peptidyl transferase centre (PTC), the progressively growing nascent chain must traverse the narrow exit tunnel^6^, which physically limits extensive intramolecular contacts, although helical formation^7^, overall compaction^8–10^ and, near the wider vestibule, small tertiary motifs have been observed^11,12^. Therefore, most proteins acquire their native structures outside the exit tunnel. However, their conformational preferences remain biased by steric occlusion^13^ and interactions with the highly charged ribosome surface^5,14–17^, which can influence their folding kinetics^18^, folding onset^13,17^, assembly^19^ and propensity to misfold^20^. The nascent chain may be further guided towards its native state by the presence of co-translational folding intermediates, as inferred from force-based assays^12^ and from the detection of generally compacted states by fluorescence-based^8,9^, optical tweezer^18^ and cysteine modification experiments^20^. However, in contrast to highly detailed studies of protein folding off the ribosome^21^, direct measurements of co-translational folding intermediates are lacking because of the substantial technical challenges associated with the flexible nascent chain tethered to a ~2.3 MDa ribosome.

Solution-state NMR spectroscopy has permitted the high-resolution characterization of ribosome-nascent chain complexes (RNCs)^5,14,15,22–26^. Here, we expand this approach by developing ^19^F NMR for co-translational folding studies, exploiting improvements in site-selective in vivo incorporation of non-canonical amino acids^27^ and the high spectroscopic sensitivity of the ^19^F nucleus^28^, which has led to a recent resurgence in its use in complex biological systems^29^. We find that this strategy permits direct, background-free observation of the co-translational folding transition and the detection of two folding intermediates of the FLN5 immunoglobulin-like domain of the multi-domain filamin FLN^15,30^. We then use molecular dynamics (MD) simulations to produce potential models of the intermediates, which corroborate previously obtained cryo-electron microscopy (cryo-EM) densities^31^ and enable rational design of mutant nascent chains to disrupt a ribosome-binding site that stabilizes their formation. These observations reveal how the ribosome can alter the folding pathway by promoting partially folded intermediates during translation.

## Results

### In vivo production of site-selective ^19^F-labelled RNCs

**Fig. 1:**
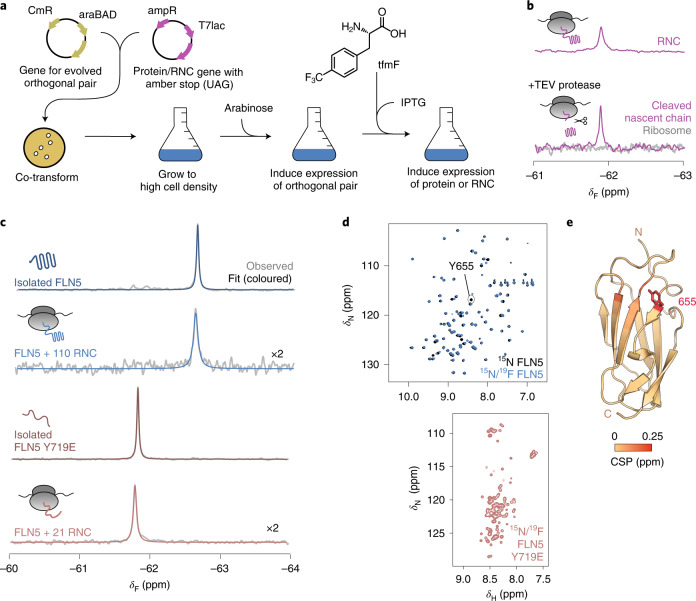
Site-specifically ^19^F-labelled RNCs report on the folding of FLN5 on and off the ribosome. **a**, Schematic of production of ^19^F-labelled RNCs (Methods). CmR, cloramphenicol resistance gene; araBAD, l-arabinose operon; ampR, ampicillin resistance gene; T7lac, T7 promoter inducible by isopropyl ß-d-1-thiogalactopyranoside (IPTG). **b**, The ^19^F NMR spectra of a RNC with a cleavable FLN5 domain, before and after addition of tobacco etch virus (TEV) protease and purification of component parts (Extended Data Fig. 1). **c**, The ^19^F NMR spectra of isolated FLN5 and FLN5 + 110 RNC, and isolated FLN5 Y719E and FLN5 + 21 RNC. Observed and fitted spectra are shown in grey and red/blue respectively (298 K, 500 MHz). *δ*_F_, 9F chemical shift. RNC spectra magnified by a factor of ×2. **d**, The 2D ^1^H,^15^N NMR (selective optimized flip angle short transient (SOFAST) heteronuclear multiple quantum coherence (HMQC)) spectra of ^15^N-labelled and ^15^N/^19^F-labelled isolated FLN5 and FLN5 Y719E (298 K and 283 K, respectively; 800 MHz). *δ*_N_, ^15^N chemical shift; *δ*_H_, ^1^H chemical shift. **e**, Crystal structure of FLN5 (Protein Data Bank (PDB) no. 1QFH) coloured by residue-specific ^1^H,^15^N amide backbone chemical shift perturbations (CSP) observed following ^19^F incorporation at position 655 (Extended Data Fig. 3). The N and C termini are shown. Source data

**Extended Data Fig. 1:**
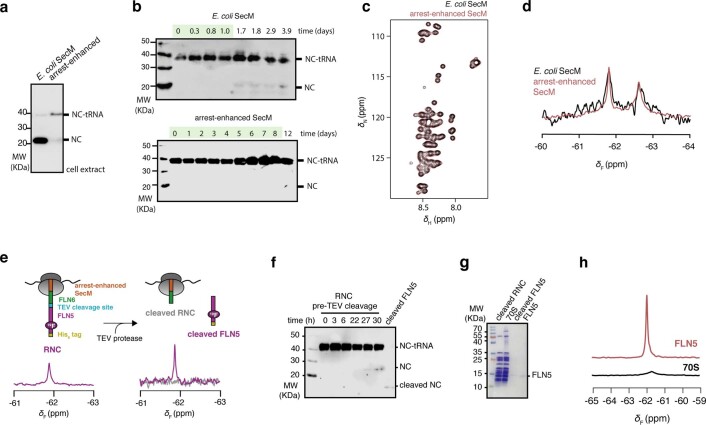
Development of *in vivo* site-selective ^19^F-labelling of arrest-enhanced RNCs using amber suppression. **a**, Anti-histidine western blot of cell extracts following expression without further purification of FLN5 + 31 RNC translationally stalled using SecM deriving from *E. coli*, and an arrest-enhanced variant of SecM based on the sequence deriving from *Mannheimia succiniciproducens*^66^ with the sequence ‘FSTPVWIWWWPRIRGPP’. A higher amount of released nascent chain relative to ribosome-bound (that is tRNA-bound) nascent chain (NC-tRNA) is interpreted as higher ribosome turnover/read-through, and thus weaker translation arrest. **b**, Anti-histidine western blot of samples of purified FLN5 + 31 A_3_A_3_ RNC^17^ with *E. coli* SecM (upper) and arrest-enhanced SecM (lower) incubated at 10˚C. Green shading indicates time during which exclusively ribosome-bound (tRNA-bound) nascent chain is detected. **c**, 2D ^1^H,^15^N-SOFAST HMQC spectra of a non-ribosome interacting FLN5 + 31 RNC variant with E. coli SecM (black) and arrest-enhanced SecM (red); no discernible difference was found. **d**, 1D ^19^F NMR spectra of FLN5 + 34 RNC with E. coli SecM (black) and arrest-enhanced SecM (red). No discernible difference was found, notwithstanding the significantly higher effective signal-to-noise provided by longer available data acquisition of the arrest-enhanced RNC. **e**, (left) 1D ^19^F spectrum of FLN5, ^19^F-labelled at position 691 and translationally stalled by the arrest-enhanced SecM motif, linked together with a linker comprising FLN6 residues and a TEV protease cleavage site. (right) 1D ^19^F spectrum following cleavage by TEV protease and purification of the two component parts to produce the cleaved RNC and cleaved FLN5. **f**, Anti-histidine western blot of RNC sample during NMR data acquisition and following TEV protease cleavage. **g**, Coomassie-stained SDS-PAGE of purified samples. **h**, 1D ^19^F spectra of purified (upper) FLN5, and (lower) 70S ribosomes purified from *E. coli* transformed with the plasmid encoding the orthogonal pair and exclusively grown in cultures supplemented with tfmF to achieve 100% tfmF labelling. Spectra are normalised to molar concentrations and number of experimental scans. These data demonstrate that even with 100% background labelling of the ribosome, its signal intensity remains substantially lower than that of FLN5. Western blots and gels show representative data from two independent repeats. Source data

**Extended Data Fig. 2:**
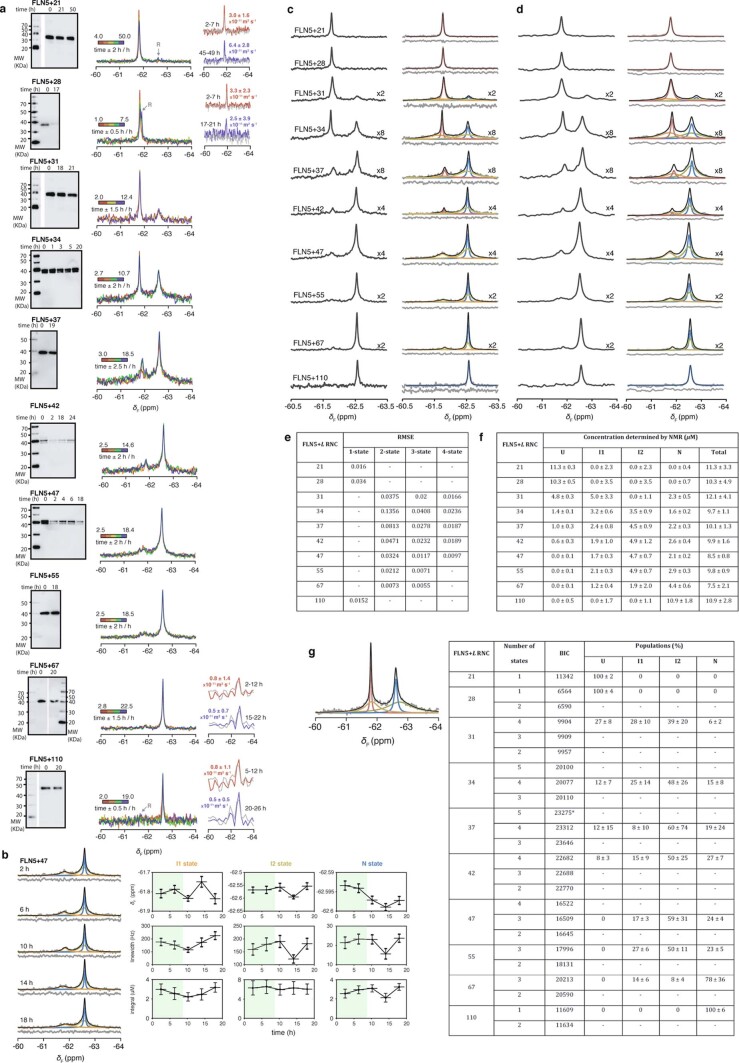
Assessment of sample integrity and lineshape fitting of FLN5 RNCs. **a**, For each RNC construct, the sample was subjected to (left) anti-histidine western blot analysis following SDS-PAGE of aliquots of a sample incubated in parallel to NMR experiments, evaluated by the observation of the tRNA-bound (that is ribosome-bound) form of the nascent chain, representative data shown from two independent repeats; (middle) an assessment of its 1D ^19^F NMR spectra recorded in timed succession; and where sensitivity was permissible, (right) ^19^F STE experiments were recorded, in an interleaved manner with 1D ^19^F experiments, with a diffusion delay of 100 ms and at gradient strengths of 5% (coloured) and 95% (grey) of the maximum gradient strength G_max_ of 0.54 T m^-1^, and summed to gain sufficient signal-to-noise to determine its diffusion coefficient. **b**, As a representative example of the assessment of 1D ^19^F NMR spectra over time, (left) spectra of FLN5 + 47 are shown (grey), fitted to Lorentzian lineshapes (coloured), with residuals after fitting shown below. (right) Quantitative analysis of the chemical shift, linewidth and integrals for each RNC state, taken from fittings of spectra over time; green shading indicates the time in which the RNC sample was deemed to be stable and intact. Data from these times were summed together and used for the final spectrum. Error bars indicate errors calculated from bootstrapping of residuals from NMR line shape fittings. 1D ^19^F NMR spectra of the FLN5 RNCs were fitted to line shapes using exponential line broadening functions prior Fourier transformation to compare spectroscopic sensitivity of broad lines (increases with stronger line broadening) and resolution between different peaks (improves with weaker line broadening). Shown in the figure are exponential line broadenings at **c** 10 Hz, and **d** 40 Hz. Analysis using either exponential function results in the same quantifications. **e**, Root-mean-square errors (RMSE) obtained for the fitting of different numbers of lineshapes to 1D ^19^F NMR spectra of the FLN5 RNCs. **f**, Concentrations of each state were determined by lineshape fitting of spectra, and normalised to a sample concentration of 10 µM as measured by its absorbance at a wavelength of 280 nm, and to which the total summed NMR integral was compared against. No significant deviation was found between the concentration determined by NMR integration and by absorbance, indicating that the lineshape fits did not significantly over- or underfit the data. **g**, Time domain analysis of FLN5 RNCs of varying lengths. NMR data, shown in Fig. 2, were fitted in the time domain using exponential functions, combined with fits for zero-order phase and baseline correction in the frequency domain. An example of NMR data fitted using time domain analysis is shown (in the frequency domain, that is following Fourier transformation). The Bayesian information criterion (BIC) value was calculated for each RNC, as an indication of the number of resonances, and thus states, which are most likely to represent the data. The model with the lowest BIC was chosen for analysis in the frequency domain. (*) Fitting with an additional state accounting for <1.5% population. Populations determined for each state by time domain analysis are consistent with those obtained by frequency domain analysis. Source data

To explore co-translational folding at high sensitivity by ^19^F NMR spectroscopy, we used the non-canonical amino acid 4-trifluoromethyl-l-phenyl alanine (tfmF), exploiting the three-fold degeneracy of the ^19^F nucleus within its rotationally mobile CF_3_ group^32^. Using an evolved orthogonal amber suppressor transfer RNA (tRNA)/aminoacyl-tRNA synthetase pair^33,34^, a single tfmF residue was biosynthetically incorporated into the FLN5 sequence by adapting our previously described protocol for in-frame amber suppression (Fig. 1a and Methods)^22,23^. In addition, an arrest-enhanced variant of the SecM motif was developed (Extended Data Fig. 1) to stall translation at a specified position and thereby produce homogenous samples of ^19^F-labelled RNCs that remained stable for the duration of NMR data acquisition, as confirmed by western blot analysis and ^19^F NMR measurements of translational diffusion (Extended Data Fig. 2). The one-dimensional (1D) ^19^F NMR spectrum of FLN5 RNC showed a single resonance, which, following selective proteolysis to release the FLN5 domain, was retained in the NMR spectrum of the cleaved nascent chain component (Fig. 1b and Extended Data Fig. 1). By contrast, the purified, parent ribosome did not produce a detectable ^19^F NMR signal (Fig. 1b), confirming the background-free and high selectivity of ^19^F incorporation by amber suppression.

### Detecting folding on the ribosome using ^19^F NMR

**Extended Data Fig. 3:**
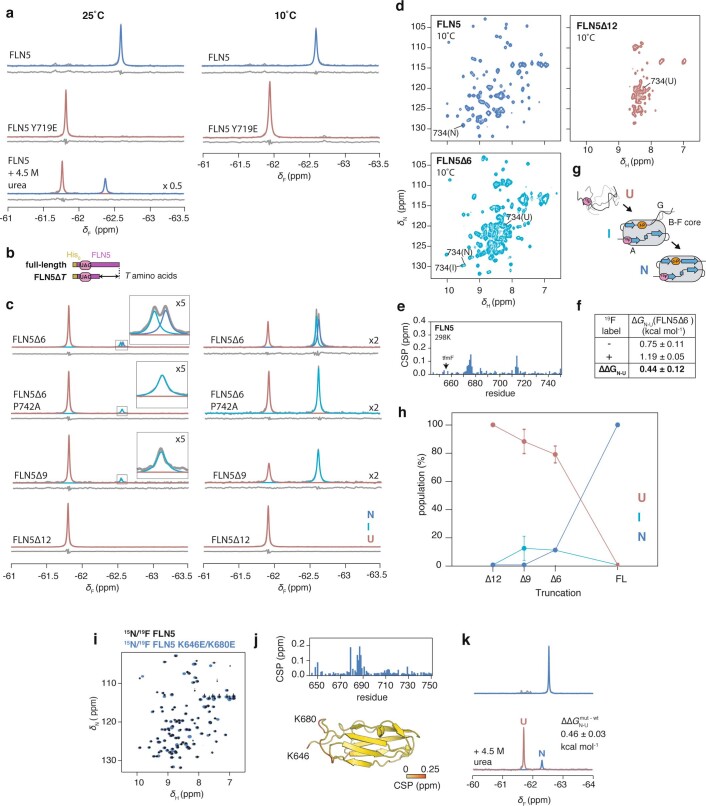
NMR spectroscopy of isolated variants of ^19^F-labelled FLN5. **a**, 1D ^19^F NMR spectra of isolated FLN5, FLN5 Y719E and FLN5 in the presence of 4.5 M urea, recorded at 25˚C and 10˚C. No intermediate state population (>5%) was detectable under denaturing conditions. **b**, Design of C-terminal truncations of FLN5. **c**, 1D ^19^F NMR spectra of FLN5∆6, FLN5∆6 P742A, FLN5∆9, and FLN5∆12, recorded at 25˚C and 10˚C. Observed spectra (in grey) were fitted to Lorentzian line shapes and assigned to the various isolated states: natively folded (N, blue), intermediate (I, cyan), and unfolded (U, red). Residuals after fitting are shown below each spectrum. The P742A mutation results in destabilisation of the ∆6 N state, enabling us to attribute the intermediate state as having a cisP742 conformation^30^. **d**, 2D ^1^H,^15^N-SOFAST HMQC spectra of FLN5, FLN5∆6, and FLN5∆12, recorded at 10˚C. Assignment of residue R734 is shown as an example of resonances in N, I, and U states. **e**, ^1^H,^15^N SOFAST-HMQC chemical shift perturbations of isolated FLN5 following introduction of Y655tfmF, at 298 K. **f**, Incorporation of tfmF results in a small destabilisation in the natively folded state, as determined by integration of FLN5∆6 peak shown in **c**, and compared against previous measurements of non-fluorinated protein^30^. Errors propagated from bootstrapping of residuals from NMR line shape fittings. **g**, Schematic summarising length-dependent folding pathway of isolated FLN5^30^. **h**, Length-dependent folding of isolated, ^19^F-labelled FLN5, determined by integration of spectra shown in **a** and **d**. Error bars indicate errors propagated from bootstrapping of residuals from NMR line shape fittings.**i**, ^1^H,^15^N-correlated NMR spectra of isolated ^15^N/^19^F-labelled FLN5 and FLN5 K646E/K680E. **j**, ^1^H,^15^N- chemical shift perturbations of FLN5 upon introduction of K646E/K680E mutations from analysis of spectra shown in **a** shown per residue in the plot, and coloured onto the FLN5 crystal structure below. **k**, ^19^F NMR spectra of FLN5 K646E/K680E in 0 and 4.5 M urea. The folded/unfolded populations of the latter were determined by lineshape fitting, and compared against those obtained for wild-type FLN5 (shown in **a**) to obtain its change in thermodynamic stability. Source data

To test the ability of ^19^F NMR to distinguish different conformations of FLN5, we examined the conservative substitution of a solvent-exposed tyrosine residue to tfmF at position 655 on β-strand A, where the nascent chain in its disordered conformation does not significantly interact with the ribosome and thus remains sufficiently dynamic for NMR observation^17^. We initially produced isolated FLN5, labelled uniformly with ^15^N and site-selectively with ^19^F at position 655, and assessed the impact of fluorination. Minimal changes in thermodynamic stability (difference in Gibb's free energy (∆∆*G*﻿) ≈ +0.4 kcal mol^−1^; Extended Data Fig. 3) and ^1^H,^15^N-correlated chemical shift perturbations (∆*δ*_HN_ < 0.15 ppm; Fig. 1d,e and Extended Data Fig. 3) were observed. The absence of the Y655 resonance in the fluorinated protein ^1^H,^15^N spectrum (Fig. 1d) confirmed the high tfmF incorporation efficiency (>95%).

The ^19^F NMR spectrum of FLN5 showed a single resonance as expected (Fig. 1c and Extended Data Fig. 3). Similarly, the ^19^F spectrum of natively folded FLN5 + 110 RNC, in which FLN5 is tethered to the ribosome by 110 linking residues^15^, contained a single peak with an identical chemical shift (Fig. 1c and Extended Data Fig. 2). A shorter linker of 21 residues (FLN5 + 21 RNC) shifts the ^19^F NMR peak by +0.8 ppm (Fig. 1c and Extended Data Fig. 2), a similar chemical shift to that of the isolated, unfolded variant of FLN5, having the Y719E point mutation (Fig. 1c,d; ref. ^15^). The chemical shift of tfmF655 is therefore a simple, direct reporter of the folding of FLN5, both on and off the ribosome.

### Identification of co-translational intermediates populated during biosynthesis

**Fig. 2:**
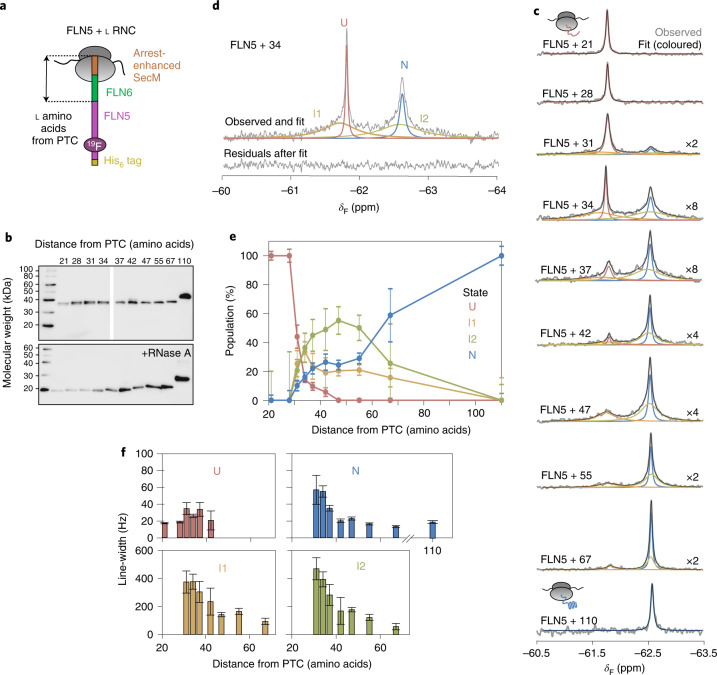
Co-translational folding of FLN5 monitored by ^19^F NMR spectroscopy. **a**, Design of FLN5 RNCs in which FLN5 is tethered to the PTC via a linker sequence comprising a variable number of FLN6 residues and an arrest-enhanced SecM stalling motif. **b**, Anti-hexahistidine western blot of purified FLN5 RNCs, with and without ribonuclease A (RNase A) treatment. Representative data shown from two independent repeats. **c**, The ^19^F NMR spectra of FLN5 RNCs with increasing distance from the PTC. Observed spectra shown in grey were fitted and peaks assigned to U, I1, I2 or N states (coloured), with the sum of the fits shown in black. NMR data were multiplied with an exponential window function (10 Hz line broadening factor) before Fourier transformation. **d**, The ^19^F NMR spectrum of FLN5 + 34 RNC, processed with a line broadening factor of 5 Hz. Residual spectrum after fitting is shown below. **e**, Folding of FLN5 on the ribosome, measured using ^19^F NMR line-shape fits. **f**, Line-widths measured by line-shape fits of spectra as shown in **c**. All error bars indicate errors calculated by bootstrapping of residuals from NMR line-shape fittings. Source data

The co-translational folding of FLN5 has previously been examined by specifically measuring its unfolded and folded state NMR resonances using ^15^N labelling and selective ^13^C-methyl labelling, respectively^15^. We explored whether ^19^F NMR could be used to directly observe the folding transition, and so produced eight additional ^19^F-labelled FLN5 RNCs, varying the number of linking residues deriving from the subsequent FLN6 domain (Fig. 2a,b and Extended Data Fig. 2; ref. ^15^), with each reporting as a representative biosynthetic snapshot at equilibrium.

The nascent chain remains unfolded with linker lengths of 21 and 28 residues (Fig. 2c). However, within the ^19^F spectra of longer RNCs (FLN5 + 31 to FLN5 + 67), we observed multiple peaks that altered in their apparent line-widths and signal intensities, indicative of a folding transition (Fig. 2c,d and Extended Data Fig. 2). Analysis of the spectra, in both the frequency and time domains, showed that FLN5 populates four distinct states during co-translational folding (Fig. 2c,d and Extended Data Fig. 2). The peak integrals are directly related to the concentrations of each state (and thus the total integral to the sample concentration; Extended Data Fig. 2) and so were used to quantify their relative populations (Fig. 2e).

The sharpest peak at −61.8 ppm, corresponding to the unfolded state (denoted U), is found in the spectra of RNCs with linker lengths of 21 to 42 residues (Fig. 2c). However, its population begins to significantly reduce beyond 28 linking residues from the PTC (Fig. 2e). Concurrently, a slower progressive increase in natively folded FLN5 (denoted N, at −62.6 ppm) is found from FLN5 + 31 to FLN5 + 110 RNCs (Fig. 2c,e). These data are consistent with previous observations of U and N by two-dimensional (2D) ^1^H,^15^N-correlated and ^1^H,^13^C-correlated NMR spectroscopy, respectively^15^.

The ^19^F NMR observations also reveal large populations of two putative intermediate states that have previously not been observed, to the best of our knowledge^15^. These states are detected as broad peaks, which persisted for the duration of the NMR experiments (Extended Data Fig. 3). The intermediates have chemical shifts similar to those of U and N, indicating the absence and presence of native-like tertiary contacts local to the ^19^F labelling site within these states, denoted I1 and I2, respectively (Fig. 2c). They are initially populated at 31 residues from the PTC (Fig. 2c), at which there is complete emergence of FLN5 from the exit tunnel^15^. I1 is maximally populated with 31–34 linking residues, while I2 is increasingly populated up to ~47 residues from the PTC before progressively reducing with linker length (Fig. 2e).

**Extended Data Fig. 4:**
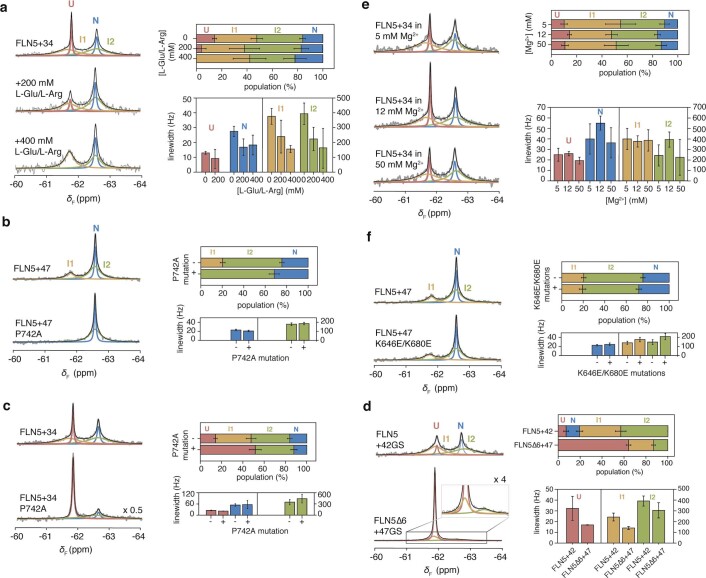
Characterisation of co-translational folding intermediates. **a**, 1D ^19^F NMR spectra of FLN5 + 34 in the presence of increasing concentrations of equimolar arginine glutamate. Line shape fittings were used to determine the populations and line widths, as shown by the plots on the right. Reductions in linewidths are indicative of loss of ribosome interactions. Decreased population of U is consistent with destabilisation of its ribosome interactions^17^. **b**, 1D ^19^F NMR spectra of FLN5 + 47 and FLN5 + 47 P742A. Line shape fittings were used to determine the populations and line widths, as shown by the plots on the right. **c**, As in **b** but with FLN5 + 34 and FLN5 + 34 P742A. **d**, As in **b** but with FLN5 + 42GS and FLN5∆6 + 47GS (283 K, 500 MHz). **e**, As in **b** but with FLN5 + 34 in 5, 12, and 50 mM magnesium ion concentration. **f**, As in **b** but with FLN5 + 47 and FLN5 + 47 K646E/K680E. Similar populations are obtained for the RNC despite the destabilisation of the native state in isolation, indicating an effective stabilisation of N on the ribosome. All error bars indicate errors (propagated) from bootstrapping of residuals from NMR line shape fittings. Source data

**Extended Data Fig. 5:**
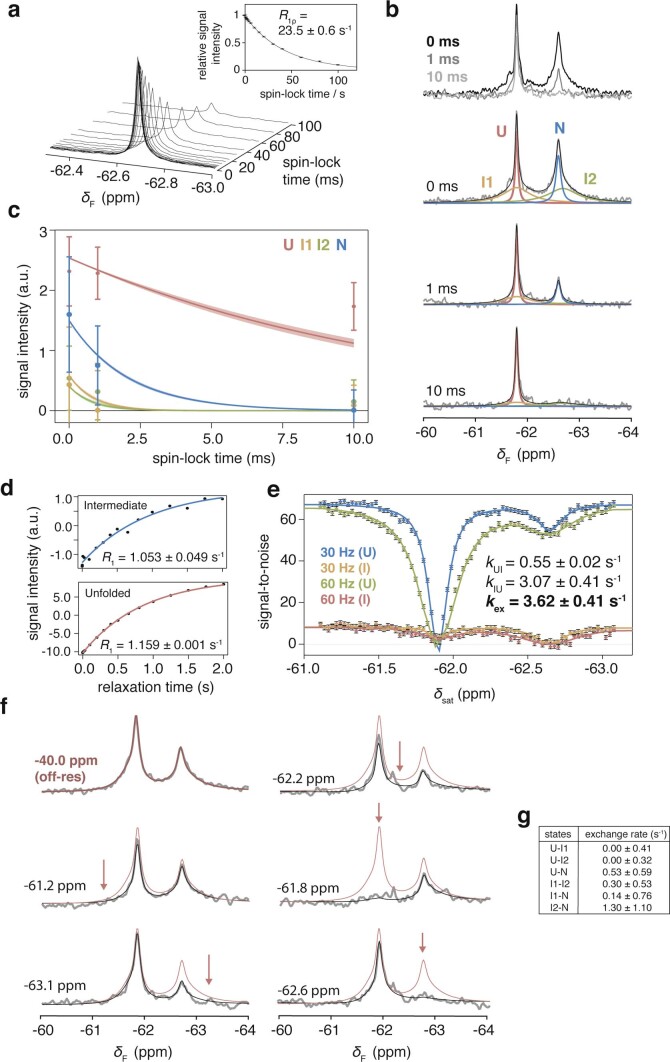
Characterisation of dynamic processes on and off the ribosome by ^19^F NMR spectroscopy. **a**, On-resonance ^19^F R1ρ measurements of isolated FLN5 using a high spin-lock field (7500 Hz). Inset shows plot of relative signal intensities from fitted spectra as a function of spin-lock time. The R1ρ determined is consistent with R_2_ measured using lineshape analysis of the 1D ^19^F NMR spectrum (23.3 ± 0.8 s^-1^, Fig S3), indicating the absence of chemical exchange processes. **b**, On-resonance ^19^F R1ρ measurements of FLN5 + 34 RNC. Due to limitations in sensitivity, we selected three spin-lock times. Observed spectra are shown above. Spectra shown below were globally fitted, with shared chemical shifts and linewidths, but independent signal intensities. **c**, Signal intensities determined from a global fit of spectra shown in **b** were plotted against spin-lock times, and compared against the expected signal decay from R_2_ measurements determined by lineshape analysis of the 1D ^19^F NMR spectrum as shown in the shaded regions. Error bars indicate errors determined by bootstrapping of residuals from NMR line shape fittings. **d**, ^19^F longitudinal relaxation rate (*R*_1_) measurements for the unfolded and intermediate states in FLN5∆6 P742A, used in the CEST measurement fittings. Error determined from data fits**. e**, ^19^F CEST profiles for FLN5∆6 P742A measuring exchange between the unfolded and isolated intermediate states using different *B*_1_ field strengths (30, 60 Hz). Error determined from data fits. **f**, ^19^F CEST measurements of FLN5 + 34 RNC. Due to limitations in sensitivity, we selected six frequencies at which to irradiate (of which one was off-resonance from all NMR peaks and shown in red, with remaining irradiation frequencies indicate d by arrows) with the 15-Hz *B*_1_ field. The frequencies were chosen to either saturate N/U states and intermediates (-62.2, -61.8, and -62.6 ppm), or only one intermediate state (-61.2 and -63.1 ppm). In the case of the latter, saturation of I1 (that is at -61.2 ppm) did not result in significant perturbation of the I2 state, and vice versa; this result indicates that the I1 and I2 resonances are distinct, in slow exchange, and therefore provides further evidence that four states are populated by FLN5 + 34. Observed spectra (grey) were fitted (black) by analysing in the time domain using the Bloch-McConnell equations. **g**, Exchange rates between FLN5 + 34 nascent chain states determined by CEST measurements, using an estimated *R*_1_ of 1.1 s^-1^ for all RNC states. Error determined from data fits. Source data

NMR peak line-widths can provide information on dynamic processes, reporting on processes such as chemical exchange and rotational tumbling^35^. To assess the effect of chemical exchange between the nascent chain states on the observed NMR line-widths, we acquired ^19^F on-resonance rotating-frame relaxation rate (R1ρ) measurements^36^ of FLN5 + 34 RNC (Extended Data Fig. 5); these data show that the I1 and I2 resonances are not the result of broadening of the U or N peaks. Line-widths are also affected by tumbling; in addition to structural conformations, line-widths of nascent chain resonances are therefore particularly sensitive to even transient, weak binding to the large ribosomal particle^5,17^. The line-widths of U remain generally sharp across all RNC lengths, indicating that the nascent chain remains mobile, at least locally to the ^19^F labelling site (Fig. 2f; ref. ^15^). By contrast, the N resonances are broad at short RNC lengths but narrow away from the ribosome (Fig. 2f) and can be attributed to faster tumbling of the globular FLN5 domain as it is extruded^25^. The line-widths of I1 and I2 are significantly broader than those of U and N (Fig. 2f), but progressively narrow with both nascent chain length (Fig. 2f) and with increasing ionic strength (Extended Data Fig. 4), indicating that they bind, partly through electrostatic interactions, to the ribosome surface, resulting in more limited mobility.

Moreover, the broad line-widths (that is, fast effective transverse relaxation rates R_2_) account for the absence of intermediate state resonances in previous NMR measurements using alternative labelling schemes; these require 2D experiments, which increases the dead time during which the signal relaxes and decays. Overall, the ^19^F NMR data identify two stable, structurally distinct intermediate states, which are populated outside the exit tunnel and are closely associated to the ribosome surface.

### Slow interconversion between nascent chain conformations

We acquired ^19^F chemical exchange saturation transfer (CEST) measurements^36^ to investigate the kinetic interconversion between the four nascent chain states. By irradiating frequencies at particular offsets from an NMR resonance with a weak applied radiofrequency (*B*_1_﻿) field, the resulting perturbation (that is, signal reduction) is transferred to the interconverting state via chemical exchange^37^. CEST measurements of FLN5 + 34 RNC (Extended Data Fig. 5) indicate that chemical exchange between all states occurs slowly (rate constant (*k*_ex_﻿) < 1.3 s^−1^, time constant ﻿(*τ*_ex_﻿) > 0.8 s). By contrast, an isolated variant of FLN5 exchanges at a faster rate of 3.6 ± 0.4 s^−1^ between its unfolded and native-like intermediate structure that lacks G-strand contacts but is otherwise folded^30^ (Extended Data Fig. 5), suggesting that the effective folding rate is reduced on the ribosome and that additional processes may potentially be competing with folding. The observed slow exchange between RNC states, corroborated by the R1ρ measurements discussed above (Extended Data Fig. 5), also verify the presence of two distinct intermediate state peaks (rather than a single, highly broadened peak), since irradiating I1 did not result in a significant perturbation of I2, and vice versa (Extended Data Fig. 5).

### Partially structured intermediates on the ribosome

Off the ribosome, truncation of the six carboxy-terminal (C-terminal) residues of isolated FLN5 (FLN5∆6) produces a population of a stable intermediate (Extended Data Fig. 3; ref. ^30^), previously characterized as having a native-like core with a detached terminal G-strand, and with the conserved *cis*-proline P742 in a *trans* conformation (Extended Data Fig. 3; ref. ^30^). Previous structural modelling has indicated that this conformation is sterically accessible on the ribosome with a linker length of at least 18 amino acids^30^, and so we sought to examine whether I1 and I2 adopted this structure.

**Fig. 3:**
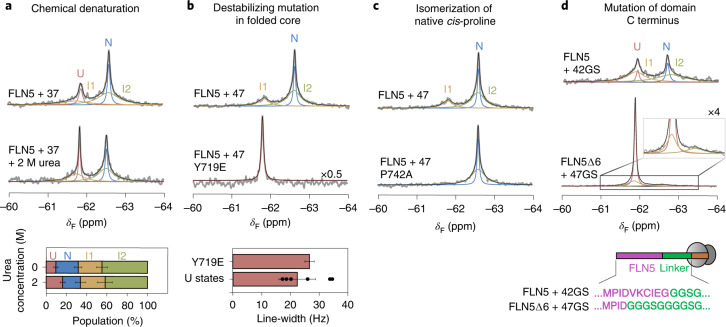
The ribosome-bound intermediate states are partially folded. **a**, The ^19^F NMR spectra of FLN5 + 37 RNC in the absence and presence of 2 M urea. Fractional populations shown below. **b**, The ^19^F NMR spectra of FLN5 + 47 and FLN5 + 47 Y719E RNC. Below, the line-width of FLN5 + 47 Y719E is compared against the line-widths of U determined for other RNCs (mean ± s.d.; Fig. 2f). **c**, The ^19^F NMR spectra of FLN5 + 47 and FLN5 + 47 P742A RNC. Analysis shown in Extended Data Fig. 4. **d**, The ^19^F NMR spectra of tfmF655-labelled FLN5∆6 + 47GS and FLN5 + 42GS RNCs (283 K, 500 MHz). Schematic depicts RNC construct design. Analysis shown in Extended Data Fig. 4. Unless stated otherwise, error bars indicate errors propagated from bootstrapping of residuals from NMR line-shape fittings. Source data

**Extended Data Fig. 6:**
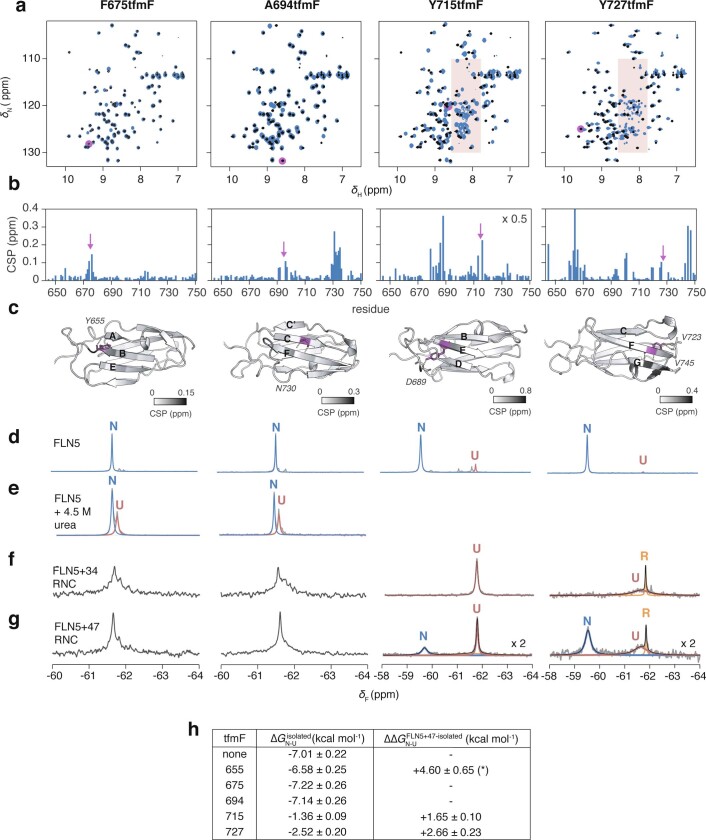
^19^F NMR spectroscopy of FLN5 with tfmF incorporation at positions F675, A694, Y715, and Y727. **a**, 2D ^1^H,^15^N-SOFAST HMQC spectra of FLN5 without (black) and with tfmF-incorporation (blue). Resonance corresponding to incorporation site is absent in each tfmF-labelled FLN5 construct, as highlighted in magenta. Red shading indicates disordered resonances resulting from destabilisation by tfmF-incorporation in solvent-inaccessible positions (see **h**). **b**, ^1^H,^15^N-correlated chemical shift perturbations measured from spectra shown in **a** upon tfmF-incorporation. **c**, Location of tfmF-incorporation (magenta) on the crystal structure of FLN5 (1qfh), coloured according to chemical shift perturbations. Contacts made by non-fluorinated FLN5 at the label site are shown by dashed lines and the contacted residues labelled. **d**, 1D ^19^F NMR spectra of tfmF-incorporated FLN5. Arrows indicate the appearance of a disordered resonance, consistent with ^1^H,^15^N-correlated NMR observations. **e**, ^19^F NMR spectra of tfmF-incorporated FLN5 incubated in 4.5 M urea, used to determine the ∆∆G of tfmF-incorporation by comparison with ^19^F NMR spectra of FLN5 labelled at position 655 and incubated in 4.5 M urea, as shown in Extended Data Fig. 5. **f**, ^19^F NMR spectra of tfmF-incorporated FLN5 + 34 RNC. **g**, ^19^F NMR spectra of tfmF-incorporated FLN5 + 47 RNC. Ribosome-released species are indicated by orange arrows. For spectra with well-resolved resonances, the data were fitted to Lorentzian line shapes. The broad linewidth of the unfolded state for tfmF727 FLN5 + 47 is consistent with its position in the ribosome-interacting segment of the domain^17^, and is reduced by ~25% relative to its linewidth in FLN5 + 34. The spectra of RNCs tfmF-labelled at positions 675 and 694 show highly overlapped resonances and so we were unable to accurately fit the peaks. **h**, Gibb’s free energies of tfmF-incorporated isolated FLN5, determined by quantification of native state peak integrals of spectra shown in **d** and **e**, and free energy differences (∆∆G_N-U_) between the ribosome-bound (with 47-residue linker) and isolated native states. The ∆∆G_N-U_ for tfmF655-labelled FLN5 (labelled *) is estimated based on a population of U determined from the spectral noise. ^19^F-labelling at positions 715 and 727 show reduced destabilisation of N on the ribosome relative to when labelled at position 655 for FLN5 + 47 RNC (similar results are obtained when including I1 and/or I2 states); tfmF side chains in positions 715 and 727 therefore form native-like tertiary contacts before those in 655 are formed in the intermediate states, consistent with a folded core comprising the B-F strands. Source data

We first tested whether the putative co-translational intermediates possessed a stable structure by incubating ^19^F-labelled FLN5 + 37 RNC in 2 M urea (Fig. 3a). We observed a shift in the folding equilibrium towards U, while populations of I1 and I2 showed no discernible change. This indicates that the intermediates possess some stable structure that is largely resistant to mildly denaturing conditions. To assess this further, we introduced the destabilizing Y719E point mutation into ^19^F-labelled FLN5 + 47 RNC (Fig. 3b), which resulted in the collapse of its three ^19^F resonances into a single sharp peak (Extended Data Fig. 2), and in which its line-width and chemical shift are consistent with an unfolded state. Residue Y719 is natively solvent inaccessible, so the ability of a mutation to completely unfold both I1 and I2 indicates that they adopt partially folded structures. Additionally, we ^19^F-labelled FLN5 + 47 RNCs at positions natively buried in the hydrophobic core (Y715 and Y727; Extended Data Fig. 6). We found ^19^F NMR resonances attributable to a native-like structure, whose thermodynamic stabilities are higher than those found in RNCs labelled at position 655 (relative to isolated FLN5; Extended Data Fig. 6), suggesting the core is at least partially formed in the intermediates.

Within the isolated FLN5 intermediate, the native-like folded core comprises the A- to F-strands, and accordingly the ^19^F chemical shift of residue 655 (residing on the A-strand) is native-like (Extended Data Fig. 3). Therefore, based on their chemical shifts (Fig. 2c), it is likely that the A-strand on I2 is also folded onto the hydrophobic core, whereas in the I1 state, native side chain contacts between the A-strand and its neighbouring residues are absent and thus the A-strand is unlikely to be completely associated.

Next, we examined isomerization of the conserved proline within the intermediates. Using populations determined from their ^19^F NMR integrals, we measured the free energy changes upon mutation of P742 to alanine, which destabilizes the *cis* conformation (Extended Data Fig. 4; ref. ^30^). The point mutation completely destabilizes I1 (∆∆*G*_I1-U_ > 1.7 kcal mol^−1^), as indicated by the absence of its ^19^F resonance in the RNC spectra (Fig. 3c and Extended Data Fig. 4), showing that I1 possesses the native *cis*-P742. However, I2 and N are only mildly, but equally, destabilized (∆∆*G*_I2–U_ = 0.8 ± 0.2, ∆∆*G*_N–U_ = 0.9 ± 0.2 kcal mol^−1^ for FLN5 + 34; Fig. 3c and Extended Data Fig. 4), indicating they likely have the same P742 conformation. Although this destablization is less than that for isolated FLN5 (∆∆*G*_N–U_ ≈ +4 kcal mol^−1^ (ref. ^30^)), previously observed ^1^H,^13^C-methyl resonance chemical shifts of RNCs show that N adopts the *cis*-proline conformation^30^; thus additional effects on the ribosome likely mitigate the destabilizing mutation within I2 and N. Overall, in contrast to the isolated intermediate (Extended Data Fig. 3; ref. ^30^), both I1 and I2 likely possess the *cis* conformer of P742, potentially rationalizing the observed slow exchange (Extended Data Fig. 5) between U and the intermediates to enable proline isomerization to occur.

The terminal G-strand (I743 to I748) directly succeeds P742 and, as described above, is detached (after truncation) from the folded core of the isolated intermediate^30^. We thus investigated its role in co-translational folding by replacing the six C-terminal FLN5 residues with a stretch of poly(glycine–serine) residues in a RNC. We found that N was completely destabilized by the series of mutations (∆∆*G*_N–U_ > 2.3 kcal mol^−1^; Fig. 3d and Extended Data Fig. 4). However, I1 and I2 both persisted, being less destabilized (∆∆*G*_I1–U_ ≈ +1.5 ± 0.2 kcal mol^−1^; ∆∆*G*_I2–U_ ≈ +1.9 ± 0.2 kcal mol^−1^; Fig. 3d), indicating that the G-strand contributes significantly less to their overall folding stabilities. We also observe narrower I1 and I2 resonances by modifying the FLN5 C terminus, suggesting that interactions between the ribosome and this nascent chain segment are reduced (Extended Data Fig. 4). We note that the G-strand resides within a ribosome-binding segment previously identified in U by ^1^H,^15^N-correlated NMR measurements^17^.

The combined NMR data (Fig. 3) therefore show that I1 and I2 possess a folded core, in which the G-strand is likely to be at least partly detached and interacting with the ribosome, while I1 is further characterized by incomplete association of the A-strand, which has been found to also be labile in folding intermediates off the ribosome^30^.

### Corroborating structural evidence of intermediate states

**Fig. 4:**
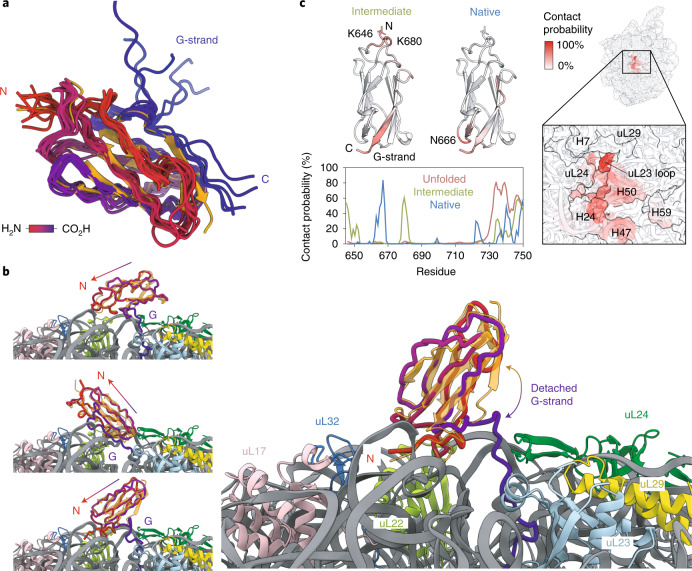
Structural ensemble of the FLN5 co-translational intermediate state determined by MD simulations. **a**, Structural ensemble of the FLN5 + 34 intermediate from CG models. The 10 most populated intermediate conformations are superimposed with the native FLN5 crystal structure (orange; PDB no. 1QFH) and coloured from N terminus (red) to C terminus (blue). Ribosome and linker are not shown for clarity. **b**, Examples of the FLN5 + 34 intermediate structures, with the FLN5 crystal structure aligned. Colours as in **a**. Arrow indicates axis from C to N terminus. **c**, The bottom left plot shows the contact probability between the FLN5 + 34 in its unfolded, intermediate and native states and the ribosome from CG models. Contact probabilities of the intermediate and native states are coloured on the FLN5 structures (above) with regions of highest probability highlighted. The right depicts the contact probability between the FLN5 + 34 intermediate and the ribosome, mapped onto the ribosome surface. Source data

**Extended Data Fig. 7:**
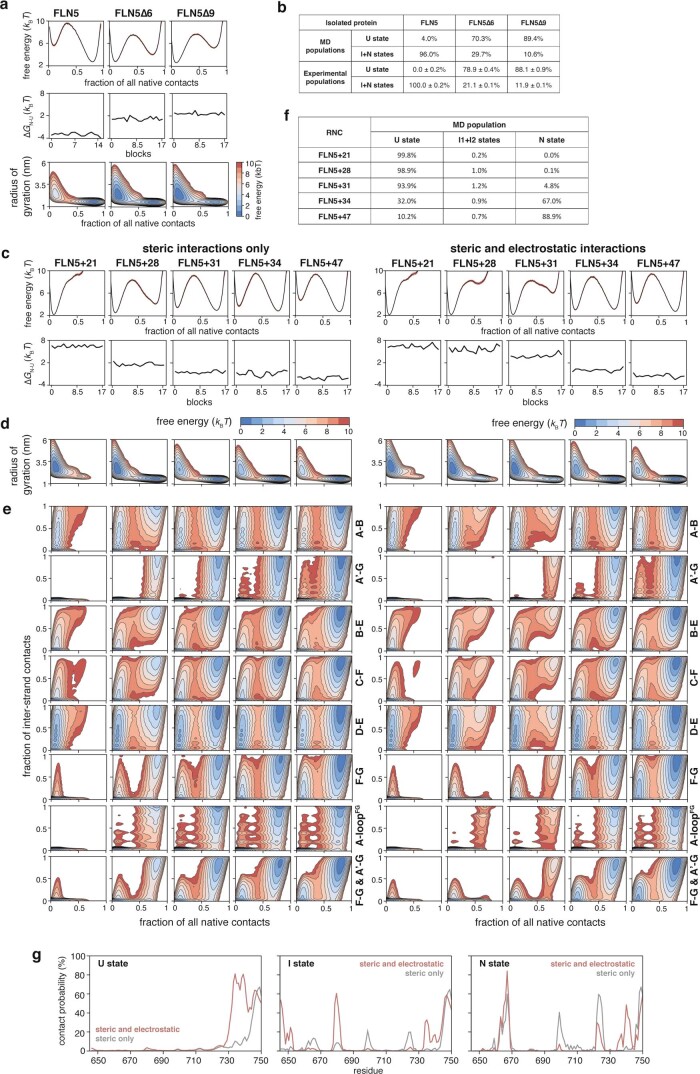
Coarse-grained molecular dynamics simulations of the co-translational folding of FLN5. **a**, CG structure-based model MD simulations of isolated FLN5 and its C-terminal truncations (FLN5∆6 and FLN5∆9) used to calibrate all subsequent MD simulations. We introduced non-bonded interactions in the form of native contacts generated from the FLN5 crystal structure (1qfh) as they dominate the folding landscape based on the principle of minimal frustration^57^. We used Parallel Biased Metadynamics^38^ to enhance sample transitions between different states using ten collective variables: fraction of all native contacts, radius of gyration, and the fraction of the native contacts between each pair of strands (A-B, A’-G, B-E, C-F, C-C’, D-E, F-F’, and F-G). Shown in the plots are the free energy landscapes of folding in 1D (top) and 2D (against the radius of gyration); the middle plot shows convergence of the free energy of folding calculated across the whole trajectory based on the block analysis. **b**, Populations of FLN5 states determined by CG models (by analysis of free energy landscapes shown in **a**) and by ^19^F NMR, showing good agreement at the chosen temperature for MD simulations. The CG models do not simulate *cis-trans* isomerisation (and thus cannot model the *trans*P742 in the intermediates^30^), and therefore, as an approximation, all folded states were compared against the summed total of native and intermediate state populations from experimental data instead. **c**, Top plot shows free energy landscapes of folding determined for 6 RNCs by CG models. Bottom plot shows convergence of the free energy of folding calculated across the whole trajectory based on the block analysis. **d**, Free energy landscapes of folding plotted against radius of gyration for each RNC. **e**, Free energy landscapes of folding plotted against fraction of contacts between pairs of β-strands or loop regions (as indicated on the right of each plot) for each RNC. **f**, Populations of unfolded, intermediate and native states obtained for each RNC by the CG models. **g**, Contact probability between the unfolded, intermediate, and native states of the RNC and the ribosome from CG models with (red) and without electrostatic interactions (grey), plotted per residue. Source data

We next performed coarse-grained (CG) MD simulations using structure-based models as an orthogonal means of examining the co-translational folding of FLN5, applying parallel biased metadynamics^38^ to enhance sampling transitions between nascent chain conformations using ten collective variables (Methods). The MD simulation temperature was calibrated to match populations of isolated FLN5 and its C-terminal truncations with those determined experimentally (Extended Data Fig. 7). The introduction of previously calibrated electrostatic interactions between FLN5 and the ribosome^17^ enabled us to accurately predict FLN5 + 31, from six RNCs (across FLN5 + 21 to FLN5 + 47), as the length at which folding begins (Extended Data Fig. 7). From the simulations, we generated and analysed the folding free energy landscapes, defined by native contacts between neighbouring β-strands, to determine the folding pathway. Consistent across the RNCs is the initial formation of native contacts within the A- to F-strands (Extended Data Fig. 7), which results in an ensemble of marginally stable intermediates (Fig. 4b), collectively characterized by a native-like core with a detached, transiently associating G-strand (Fig. 4a). Despite capturing only a single, lowly populated intermediate state (Fig. 2e and Extended Data Fig. 7), the simple CG models propose structures (Fig. 4b) that are qualitatively consistent with the ^19^F NMR data of I2 (Fig. 3). The reduced contacts observed between the A-strand and its neighbouring loop region (between strands F and G) within the same structures (Extended Data Fig. 7) may account for I1 within the structural ensemble.

Contacts made by the nascent chain with the ribosome surface in the MD simulations (Fig. 4c and Extended Data Fig. 7) correlate well with previous NMR measurements: trajectories for U show strong (up to 80% contact probability), predominantly electrostatic interactions at its C-terminal binding site (residues N728–C747) and weak contacts elsewhere^17^, while contacts between N and the ribosome occur at the domain’s C-terminal hemisphere and are largely steric with only small electrostatic contributions (Fig. 4c and Extended Data Fig. 7; ref. ^25^). We find that a significant proportion (~50%) of the intermediate ensemble contacts the ribosome through charge interactions (Extended Data Fig. 7). The interactions identified (Fig. 4c) are localized at the C terminus, as observed for U although less strong, and are consistent with experimental data (Fig. 3d and Extended Data Fig. 4). Contacts are also found at the more positively charged, amino-terminal (N-terminal) hemisphere of FLN5, centred at residues K646 and K680, which preferentially orients the partially folded domain towards the RNA-rich side of the ribosome vestibule (Fig. 4b), predominantly contacting rRNA helices H24, H47 and H50 (Fig. 4c).

**Extended Data Fig. 8:**
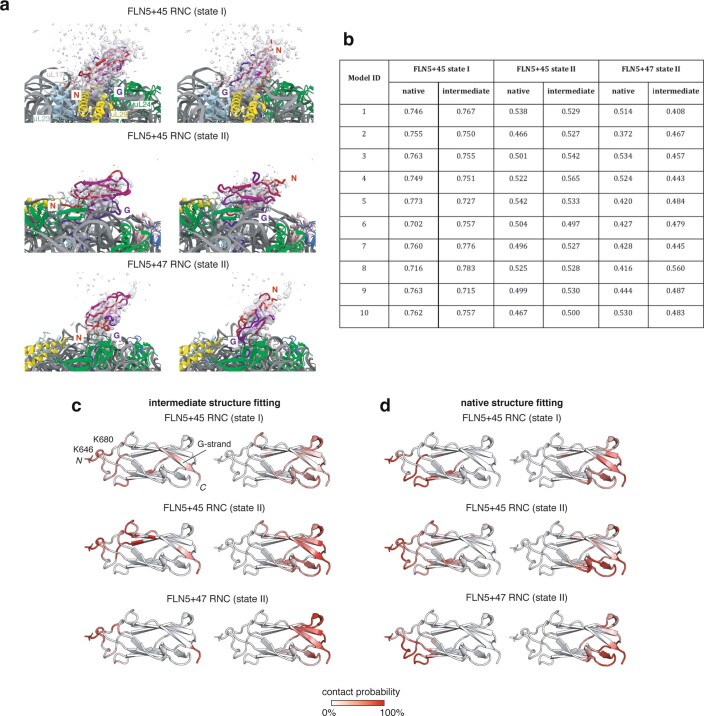
Models of the co-translational intermediates of FLN5 by all-atom, cryo-EM density-driven MD simulations. **a**, Examples of structural models of FLN5 co-translational intermediates fitted to previously obtained cryo-EM densities of FLN5 + 45 and FLN5 + 47 RNCs^31^. Two major orientations are observed, in which the N-terminus of the FLN5 domain points towards (left) or away (right) from the ribosome. The FLN5 domain is coloured from its N- (red) to C-terminus (blue), with its N-terminus (N) and G-strand labelled (G). Cryo-electron densities are shown in grey, and at a contour level of two sigma. **b**, Cross-correlation values for cryo-EM density-guided MD simulations of native and intermediate state RNCs. For each of the 10 density-guided simulations obtained using the three electron density maps^31^, we generated final models for the intermediate state, from which electron density maps were simulated and compared against the nascent chain experimental maps. The resulting cross-correlation values, calculated as in ChimeraX^65^, for the intermediate state are shown alongside cross-correlations using the same approach as previously for the native state (Javed et al, submitted). **c**, Contact probabilities of FLN5 residues with the ribosome surface by analysis of models of the intermediate as shown in **a** for the two main orientations observed (N-terminus of FLN5 towards and away from the ribosome, left and right, respectively). Regions of highest contact probability, residues K646 and K680 and G-strand, labelled. **d**, As in **b**, but for models of the native state from previous all-atom cryo-EM density-driven MD simulations using the same cryo-EM map^31^. Source data

We subsequently re-examined cryo-EM data obtained for FLN5 + 45 and FLN5 + 47 RNCs^31^, previously fitted with all-atom density-guided MD simulations with exclusively native structures defined within structure-based models. Having discovered that these RNCs predominantly populate partially folded intermediates in this work (Fig. 2), we used the previously obtained electron densities as restraints to fit structures with inter-residue contacts characterizing I2 (Fig. 4a) instead (Extended Data Fig. 8). These new models showed cross-correlations that were quantitatively similar to those obtained for natively folded structures (Extended Data Fig. 8). Additionally, the intermediate conformations also showed binding to the ribosome surface at the N-terminal loop regions and the G-strand of FLN5 (Extended Data Fig. 8), as identified in the CG models (Fig. 4c). We conclude that the cryo-EM data corroborate the proposed intermediate state structures and their interactions with the ribosome.

### Mechanism of intermediate state stabilization on the ribosome

**Fig. 5:**
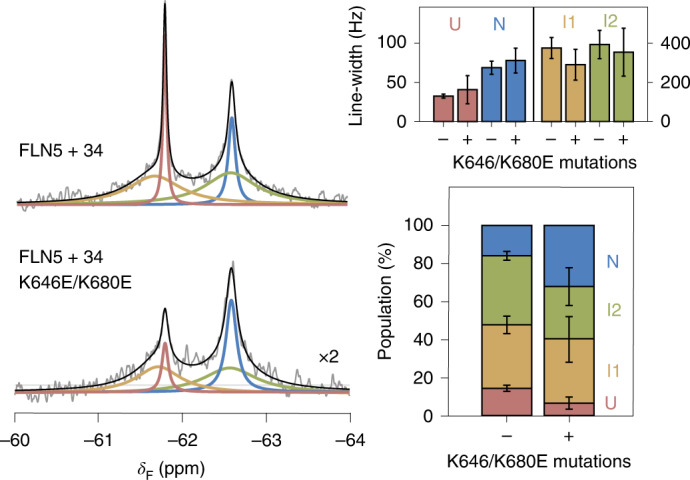
Electrostatic interactions with the ribosome surface stabilize partially folded nascent chains. The ^19^F NMR spectra of FLN5 + 34 and FLN5 + 34 K646E/K680 RNCs. Line-widths and populations of each RNC state determined by analysis of the spectra are shown on the right. Error bars indicate errors (propagated) from bootstrapping of residuals from NMR line-shape fittings. Source data

We next sought to experimentally examine the effect of the identified binding site on co-translational folding. We thus replaced residues that are predicted to strongly bind to the ribosome, K646 and K680 (Fig. 4c), found natively in the loop regions, with glutamic acid residues to reverse their charge. The ^19^F NMR spectrum of the FLN5 + 34 K646/K680E RNC shows that folding remains four-state (Fig. 5). However, the N is stabilized on the ribosome by 0.6 ± 0.3 kcal mol^−1^ relative to U, despite the mutations destabilizing the FLN5 domain off the ribosome by ~0.4 kcal mol^−1^ (Extended Data Fig. 3). Moreover, both I1 and I2 are each destabilized relative to N by 0.2–0.3 kcal mol^−1^. This shift in co-translational folding, together with a small reduction in the line-widths of I1 and I2 (Fig. 5), is therefore consistent with disruption of ribosome interactions that contribute to the stabilities of the intermediates. The folding equilibrium is also shifted towards N in a longer nascent chain possessing the same mutations (Extended Data Fig. 4), although to a lesser extent, indicating that the interactions mediated by K646 and K680 are strongest closest to the ribosome surface. However, the persistence of broad NMR resonances attributable to the intermediate states suggest that I1 and I2 possess additional stabilizing binding sites or other modes of interactions that were not defined within the CG models.

Electrostatic interactions between the nascent chain and the ribosome can also be mediated via magnesium ions^5,39^. To examine this, we analysed ^19^F NMR spectra of FLN5 + 34 RNC recorded at different concentrations of magnesium ions (Extended Data Fig. 4). In contrast to varying the overall ionic strength (Extended Data Fig. 4), we found the effect of magnesium to shift the co-translational folding equilibrium to be only very modest.

### Stabilization of partially folded nascent chains during translation

**Fig. 6:**
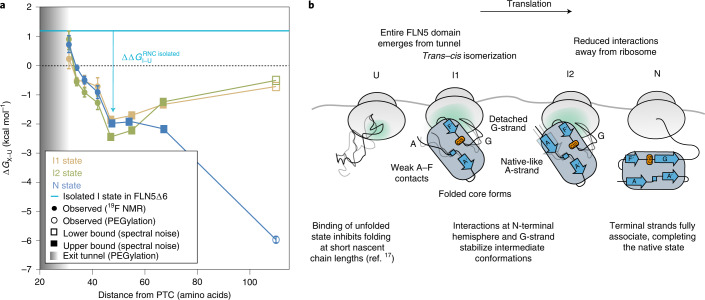
Free energy landscape and proposed model of co-translational folding of FLN5. **a**, Free energy landscape of the co-translational folding of FLN5. Fractional populations determined by ^19^F NMR (Fig. 2e) were used to determine the difference in free energy between X (I1, I2 or N) and U (∆*G*_X–U_); in RNCs where U was not populated, an upper or lower bound of ∆*G*_X–U_ was determined based on the spectral noise. The ∆*G*_N–U_ of FLN5 + 110, and emergence from the tunnel (by solvent accessibility of the native, C-terminal C747 of FLN5; shaded region) were determined by PEGylation^15,17^. Error bars indicate errors propagated from bootstrapping of residuals from NMR line-shape fittings. **b**, Schematic of a proposed model for the co-translational folding mechanism of FLN5. Source data

**Extended Data Fig. 9:**
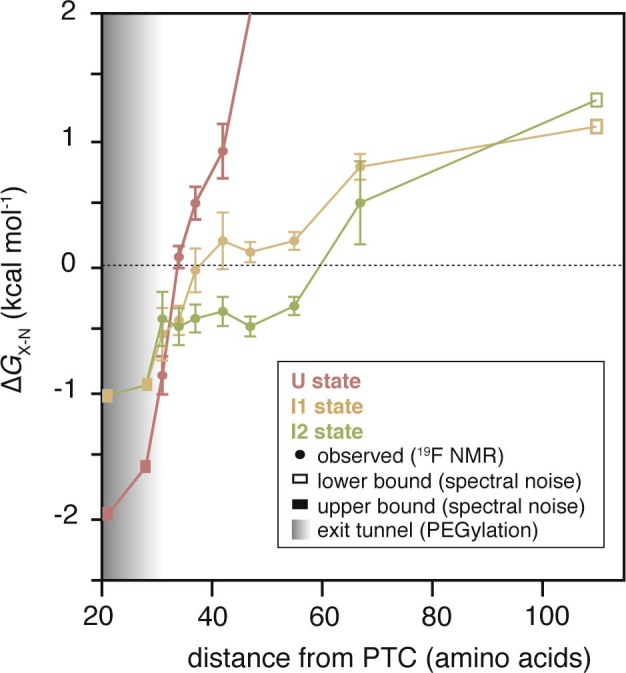
Stabilisation of co-translational intermediates by the ribosome. Free energy landscape for the length-dependent co-translational folding of FLN5, plotted relative to N, from analysis of spectra shown in Fig. 2. Error bars indicate errors propagated from bootstrapping of residuals from NMR line shape fittings. Source data

We determined a free energy landscape of the co-translational folding of FLN5 (Fig. 6a) by quantitative analysis of the RNC ^19^F NMR spectra (Fig. 2c,d). This thermodynamic analysis reveals that N is progressively destabilized close to the ribosome (Fig. 6a). Relative to N (Extended Data Fig. 9), the intermediates are more stable at short linker lengths and become progressively less stable with translation, suggesting that they are stabilized by close proximity to the ribosome. Indeed, the intermediates are substantially more stable (∆*G*_I–U_ = −2.5 to −0.2 kcal mol^−1^; Fig. 6a) at all nascent chain lengths than those found off the ribosome (∆*G*_I–U_ > +1.2 kcal mol^−1^; Extended Data Fig. 3). Folding intermediates of FLN5 are therefore stabilized on, and particularly close to, the ribosome. These observations can, at least partly, be accounted for by electrostatic ribosome interactions that selectively target and stabilize the intermediate states (Fig. 6b).

### Intermediates in co-translational multi-domain folding

**Extended Data Fig. 10:**
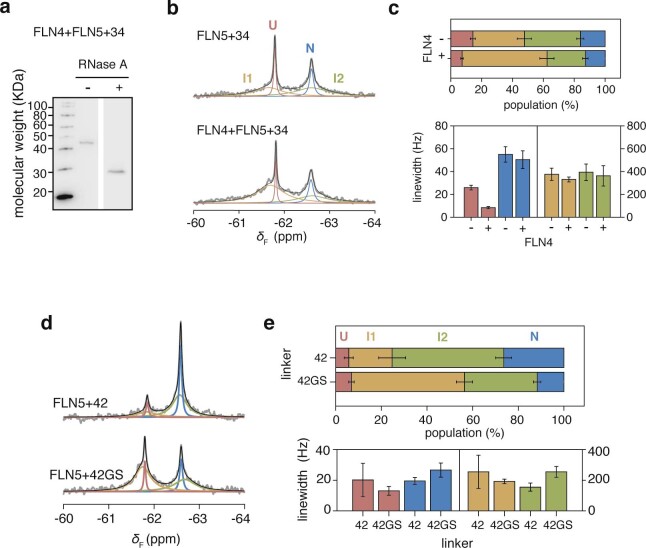
Effect of neighbouring domains on the co-translational folding of FLN5. **a**, Anti-hexahistidine western blot of the tandem FLN4 + FLN5 + 34 RNC with and without RNase A treatment. Representative data shown from two independent repeats. **b**, 1D ^19^F NMR spectra of FLN5 + 34 and FLN4 + FLN5 + 34 RNCs. **c**, Analysis of linewidths and populations from lineshape fittings of spectra shown in **b**. **d**, 1D ^19^F NMR spectra of FLN5 + 42 RNC with linker residues deriving from FLN6 and with a poly(GS) linker; the line shape fittings were used to determine the populations and line widths as shown in **e**. All error bars indicate errors (propagated) from bootstrapping of residuals from NMR line shape fittings. Source data

Finally, we considered the co-translational folding of FLN5 within the multi-domain protein. Selective ^19^F labelling of a tandem FLN4 + FLN5 + 34 RNC at the same position (residue 655) enabled a comparative analysis to assess the impact of the neighbouring FLN4 on the folding of FLN5. We observed four NMR resonances, indicating that folding remained four-state, with no significant changes in the line-width of N (Extended Data Fig. 10), the latter suggesting that the two domains tumble relatively independently from each other. At 34 residues from the PTC, we found that the presence of FLN4 increases the stabilities of I1, I2 and N (∆∆*G*_X–U_ of −0.7 to −0.2 kcal mol^−1^, where X = I1, I2 or N). To examine the effect on the folding of FLN5 of its other neighbouring domain, we replaced the FLN6 linking residues with a poly(glycine–serine) sequence in an FLN5 + 42 RNC (Extended Data Fig. 10). This resulted in destablization of both I2 and N (∆∆*G*_X–U_ of 0.4 to 0.6 kcal mol^−1^) and a stabilization of I1 (∆∆*G*_I1–U_ ≈ −0.4 kcal mol^−1^). Therefore, the data show that the neighbouring domains stabilize N and also appear to modulate the stabilities of the intermediate states of FLN5, which persist within the tandem repeat protein. This complex interplay of inter-domain interactions and ribosome binding (Fig. 5) is likely to be modulated by nascent chain length^40^, and thus may contribute to regulating multi-domain folding.

## Discussion

In this work, we have developed an experimental strategy to examine the structures, thermodynamics and kinetics of inherently heterogenous populations of nascent chains as they begin to fold outside the ribosome exit tunnel. The near-dead-time-free 1D ^19^F NMR experiments afford greater spectroscopic sensitivity relative to other isotopic labelling schemes, and thus enable detection of highly broadened resonances within spectra free of background signal. In the case of FLN5, ^19^F NMR enables direct, quantitative measurements of its co-translational intermediates that are closely associated to the ribosome surface, and their identification can provide a structural basis on which to model specific conformations within innately sparse cryo-EM densities of dynamic nascent chains. The strategy thus enables examination of the possible conformations accessible to the nascent polypeptide chain at equilibrium, and is highly amenable to other nascent chain systems, permitting expansion of RNC studies by NMR to larger, more complex multi-domain proteins.

The formation of co-translational intermediates can be regulated kinetically on the ribosome through variations in translation rate^1,41^ and stalling induced by the nascent chain^20,42^. Here, we show that the ribosome exerts a strong thermodynamic effect on the co-translational intermediates of FLN5, resulting in significantly higher stabilities relative to those off the ribosome (∆∆*G*_I–U_ ≈ +1.4–5.2 kcal mol^−1^; Fig. 6a). Moreover, a wider folding transition is observed on the ribosome (>36 versus ~12 residues off the ribosome) during which the difference in stabilities between the intermediates and N is only <1 kcal mol^−1^ (Extended Data Fig. 9). Under the quasi-equilibrium conditions in which co-translational folding occurs^9^, the wider folding transition likely enables population of the intermediates during the relative slow rate of translation. Moreover, combined with their slow interconversion rates (Extended Data Fig. 5), these observations point towards competing (not necessarily unproductive) processes that increase the energy barriers between the states. This would result in a rugged energy landscape, which could provide some resistance to external perturbations to its folding pathway. Experiments with the rationally designed charge mutants show that electrostatic interactions with the ribosome (Fig. 5) provide one mechanism by which partial folds are selectively stabilized co-translationally, although it is likely that there are other stabilizing effects, such as the presence of neighbouring domains (Extended Data Fig. 10) and hydrophobic interactions^43^. Such holdase activity has also been observed for molecular chaperones, such as the ribosome-associated trigger factor^5,44^, which assist in protein folding by promoting partial folds to narrow the nascent chain’s stochastic conformational search for its native state. Our observations therefore corroborate the view of the ribosome as the first molecular chaperone that engages the nascent chain.

In summary, our ^19^F NMR data describe how the ribosome alters the folding pathway of a nascent multi-domain protein by selectively stabilizing partially folded conformations. This has implications for our understanding of intermediates in other co-translational processes, such as misfolding^20^ and assembly^19^, and as potential druggable targets^45^.

## Methods

### Sample preparation

Using site-directed mutagenesis, amber mutations were site-specifically introduced into plasmids encoding isolated protein or RNC, the latter comprising an arrest-enhanced variant of SecM with the sequence FSTPVWIWWWPRIRGPP (Extended Data Fig. 1). After co-transformation into BL21(DE3) *Escherichia coli* with the pEVOL*-*pCNF-RS suppressor plasmid^33,34^, cells were grown using a previously described protocol^23^ with the following modifications to incorporate non-natural amino acids: cultures were supplemented with arabinose (0.2% (w/v)) to induce expression of the orthogonal pair; the EM9 expression media was further supplemented with 4-trifluoromethyl-l-phenyl alanine (1 mM) and the culture incubated for 15 min at 37 °C before addition of IPTG (1 mM) and further incubation of 1 h (RNCs) or 4 h (isolated protein). Isolated protein and RNC constructs were purified and their quality biochemically assessed as previously described^23,30^.

### NMR spectroscopy

NMR data were recorded at 298 K, unless stated otherwise, and acquired using TopSpin 3.5pl2 on a 500 MHz Bruker Avance III spectrometer (^19^F NMR) and a 800 MHz Bruker Avance III HD spectrometer (^1^H,^15^N NMR), both equipped with TCI cryoprobes. All RNC (6.4–15.0 μM) and isolated protein (100 μM) samples were prepared in Tico buffer containing 10 mM HEPES buffer, 30 mM NH_4_Cl, 12 mM MgCl_2_ and 2 mM beta-mercaptoethanol, at pH 7.5, containing 10% D_2_O and 0.001% sodium trimethylsilylpropanesulfonate. Multiple 1D ^19^F pulse-acquire experiments were recorded in succession with an acquisition time of 350 ms and a recycle delay of 3 s to ensure complete relaxation between each scan and thereby enable quantification of peak integrals. Where sensitivity was permissible, experiments were interleaved with ^19^F stimulated-echo diffusion measurements, recorded using a diffusion delay of 100 ms and 4 ms trapezoidal gradient pulses with gradient strengths of 0.027 and 0.513 T m^−1^. The 2D ^1^H,^15^N-SOFAST HMQC experiments^46^ were recorded with acquisition times of 50.4 and 29.5 ms in the direct and indirect dimensions, respectively, and a recycle delay of 100 ms. The ^19^F CEST measurements^36^ were recorded with an acquisition time of 200 ms and a recycle delay of 30 ms, with a weak *B*_1_ field of 15 Hz applied for a saturation time of 800 ms at saturation frequencies of either −40, −61.2 and −61.3 ppm, or −40, −62.2, −61.8 and −62.6 ppm. The ^19^F on-resonance R1ρ measurements^47^ were recorded using different spin-lock times with a spin-lock field of 7,500 Hz and the ^19^F frequency carrier centred at −62.6 ppm (isolated) or −62.2 ppm (RNC).

Data were processed and analysed with nmrPipe^48^, CCPN Analysis^49^, MATLAB (R2014b, The MathWorks Inc.) and Julia 1.5 (ref. ^50^). The time-domain ^19^F NMR spectra were multiplied with an exponential window function with a line broadening factor of 10 Hz, unless stated otherwise, prior to Fourier transformation. The 1D spectra were imported into MATLAB for baseline correction to eliminate background signal deriving from Teflon within the spectrometer probe, and subsequent analysis using Lorentzian functions. Reliable, quantitative measurements from line-shape fitting can be impacted by factors such as low signal-to-noise and spectral overlap; errors were therefore calculated by bootstrapping of residuals using multiple fittings^51^, and the residuals after fits were quantified. Where no resonance was observed for a state (detection level of ~5%), the error for population of the absent state was estimated from the spectral noise. The spectra were initially analysed individually (or summed with additional spectra until sufficient signal-to-noise was achieved) to assess sample integrity. Data indicating nascent chain release or sample degradation (Extended Data Fig. 2), through changes in line-widths, signal intensity or chemical shifts, were not used in the summation of spectra to produce the final spectrum, which was subjected to a final round of fitting and analysis. The number of peaks fitted to each spectrum was confirmed by a Bayesian analysis of fits performed on the NMR data in the time domain^52^. Similar populations of each state were obtained by analysis of NMR data in both the time and frequency domains.

### CG MD simulations

We used MD simulations with the Cα structure-based potential generated by SMOG 2.3 (refs. ^53,54^) to simulate the isolated FLN5 and its length variants as well as RNCs. The original CG potential is defined only for proteins, and we extended it to RNCs by describing rRNA with three beads per nucleotide and placing them at the P, C4′ and N3 atom positions^17^. Additionally, the electrostatic interactions between the ribosome and the nascent chain were introduced using Debye–Hückel theory^55^, with parameters chosen to reproduce the experimentally observed bound populations of unfolded RNCs^17^. The model of ribosome used in RNC simulations was derived from the high-resolution *E. coli* structure (PDB no. 4YBB; ref. ^56^) and consisted of the exit tunnel and ribosome surface surrounding it, which we defined based on the contact analysis from our previous simulations^17^. Atoms of the ribosome model were kept fixed during MD simulations. Each nascent chain starting structure, combining His-tag, FLN5 domain, FLN6 linker and arrest-enhanced SecM, was manually modelled inside the exit tunnel as an unfolded polypeptide chain and attached to the P-site tRNA via the SecM C-terminal proline residue, which was fixed during the simulations. Starting structures for the MD simulations of isolated full-length FLN5, as well as two truncations (FLN5∆6 and FLN5∆9), were generated from the FLN5 crystal structure (PDB no. 1QFH). The nascent chain native contacts were used in the structure-based potential as the only attractive non-bonded interactions that drive protein folding based on the principle of minimal frustration^57^, and were defined based on the FLN5 crystal structure using the OV + rCSU method^58^ and modelled with the Lennard-Jones potential. In the structure-based MD simulations (as they are set up in SMOG), we use reduced units (so the length scale, time scale, mass scale and energy scale are all equal to 1 with the only exception that the Boltzmann constant is *k*_B_ = 0.00831451, as it is hardwired in GROMACS); hence, we do not have a direct correspondence between experimental temperature and the one used to set up simulations. To mimic the experimental conditions in the MD simulations with a structure-based potential, we chose the temperature (120 K) of the simulations so that for the isolated FLN5 and both truncations (FLN5∆6 and FLN5∆9), the obtained populations are consistent with NMR observations. We used the same temperature for the RNC MD simulations.

We used an enhanced sampling method to sample the whole free energy landscape more efficiently on the ribosome. We applied Parallel Biased Metadynamics (PBMetaD^38^) with 12 walkers and with ten collective variables capturing the folding process: the ratio of the native contacts (*Q*), the radius of gyration and eight collective variables describing the ratio of the native contacts between each pair of strands: A–B, A′–G, B–E, C–F, C–C′, D–E, F–F′ and F–G. Gaussians corresponding to the bias potential were added every 2,000 steps with the height of 0.5, and the bias factor was set to 10. Simulations were run using Langevin dynamics for 3 × 108 time steps in GROMACS 4.5.7 (ref. ^59^) using PLUMED 2.6 (ref. ^60^) for introducing PBMetaD. Convergence was assessed using block analysis (Extended Data Fig. 7) and trajectories analysed using PLUMED, MDAnalysis^61^ and VMD^62^.

### All-atom electron-density-guided MD

For density-guided MD simulations, we used all-atom structure-based models generated with SMOG^53,54^ and native contacts described based on the FLN5 crystal structure; however, to fit the intermediate state, we removed contacts involving the G-strand. These MD simulations, recently introduced to GROMACS^63,64^, employ the gradient of similarity, defined using cross-correlation between a simulated density and an experimental cryo-EM density, as an additional force that is applied to atoms of the system. We used three previously published cryo-EM maps^31^ describing two states of FLN5 + 45 and one state of FLN5 + 47 RNCs. We set up ten simulations for each map, starting from different initial nascent chain positions. We used an adaptive force scaling protocol, during which the simulation slowly increases the force constant that is scaling the similarity measure (cross-correlation) in the effective potential, and thus increasing the force that drives the structure into the EM density. Finally, we stopped the simulations and selected the final structures using criteria previously described. Based on each model, we simulated its density at 10 Å resolution and compared it to the experimental cryo-EM density of the RNC using cross-correlation as defined in ChimeraX 1.4 (Extended Data Fig. 8; ref. ^65^). The cross-correlations obtained were compared against those from initial simulations with all native FLN5 contacts.

### Reporting summary

Further information on research design is available in the Nature Research Reporting Summary linked to this article.

## Online content

Any methods, additional references, Nature Research reporting summaries, source data, extended data, supplementary information, acknowledgements, peer review information; details of author contributions and competing interests; and statements of data and code availability are available at 10.1038/s41557-022-01004-0.

## Supplementary information

Reporting Summary

## Data Availability

Data supporting the findings of this study are included in the article, source data and extended data. The PDB structure 1QFH was used in this study. Source data are provided with this paper.

## Source data

Source Data Fig. 1 Source data.
Source Data Fig. 2 Source data.
Source Data Fig. 2 Unprocessed western blots.
Source Data Fig. 3 Source data.
Source Data Fig. 4 Source data.
Source Data Fig. 5 Source data.
Source Data Fig. 6 Source data.
Source Data Extended Data Fig. 1 Source data.
Source Data Extended Data Fig. 2 Source data.
Extended Data Fig. 2 Unprocessed gels and western blots.
Source Data Extended Data Fig. 3 Source data.
Source Data Extended Data Fig. 4 Source data.
Source Data Extended Data Fig. 5 Source data.
Source Data Extended Data Fig. 6 Source data.
Source Data Extended Data Fig. 7 Source data.
Source Data Extended Data Fig. 8 Source data.
Source Data Extended Data Fig. 9 Source data.
Source Data Extended Data Fig. 10 Source data.
Source Data Extended Data Fig. 10 Unprocessed western blots.

## Code availability

NMR pulse sequences are available on Github (https://github.com/chriswaudby/pp). Codes used to fit the NMR spectra are available on Guthub (https://github.com/shschan/NMR-fit).
