# Supplementary figures and images for: The ribosome stabilizes partially folded intermediates of a nascent multi-domain protein
Source: Nat Chem. 2022 Aug 4;14(10):1165–73. doi: 10.1038/s41557-022-01004-0 (PMC7613651; doi:10.1038/s41557-022-01004-0)

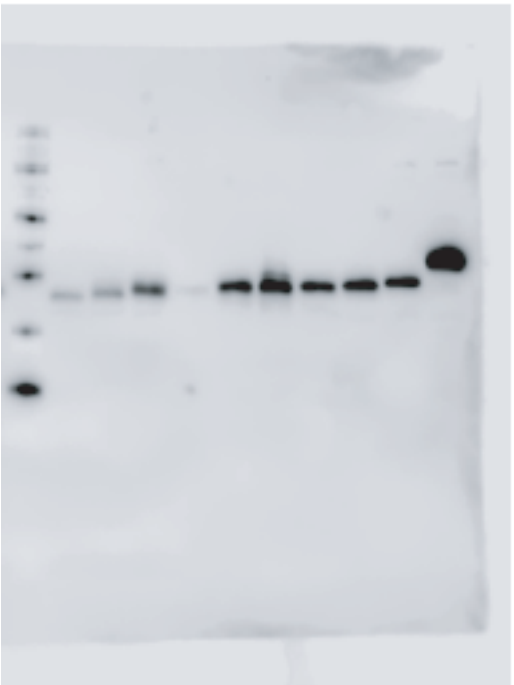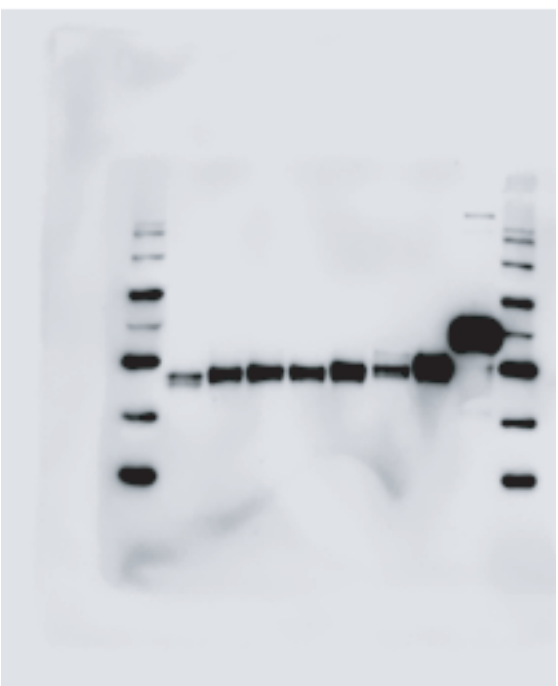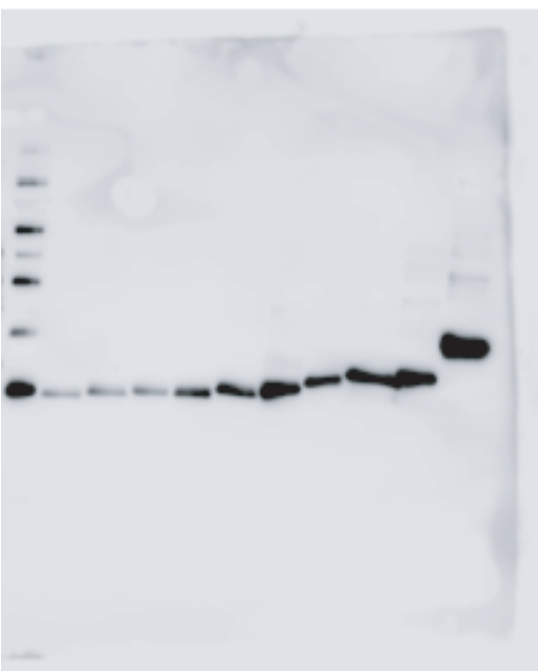

Supplement: Source Data Fig. 2 — Unprocessed western blots. [file 41557_2022_1004_MOESM4_ESM.pdf]

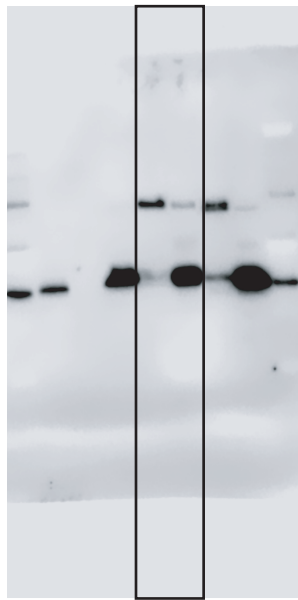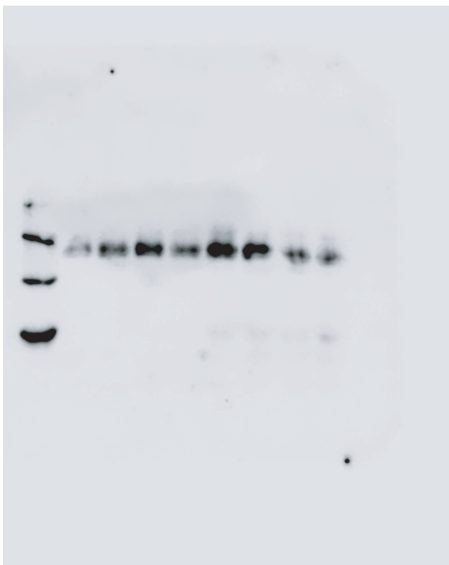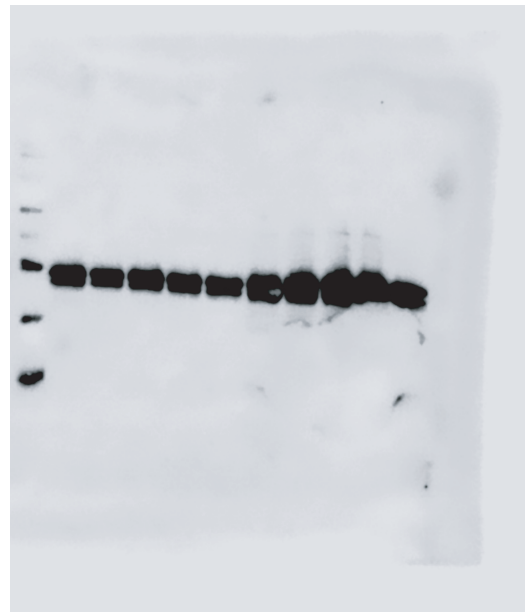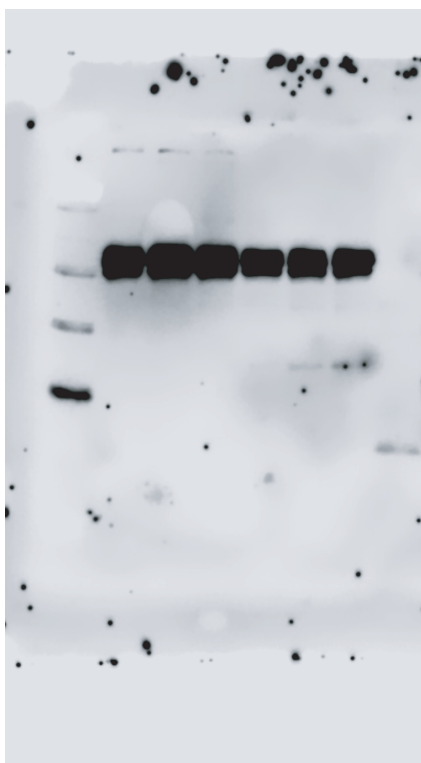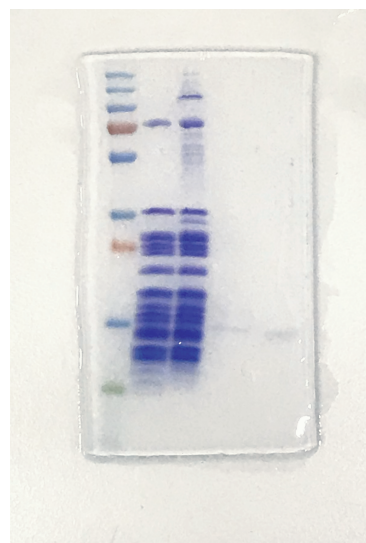

Supplement: Source Data Extended Data Fig. 1 — Source data. [file 41557_2022_1004_MOESM9_ESM.pdf]

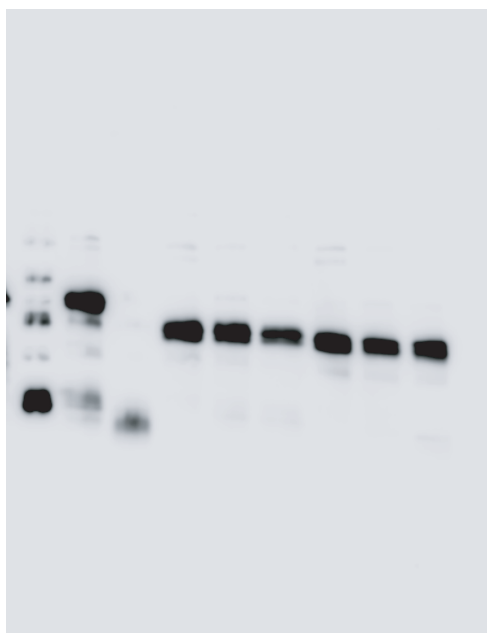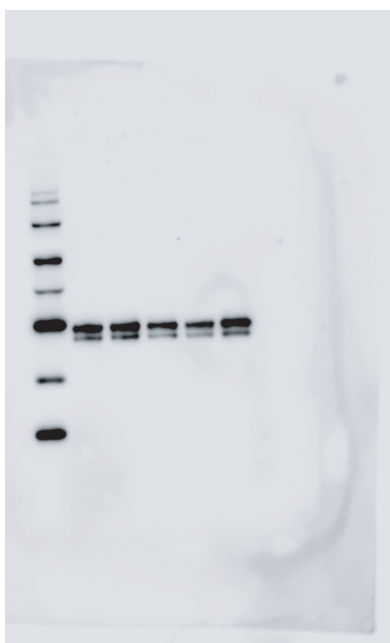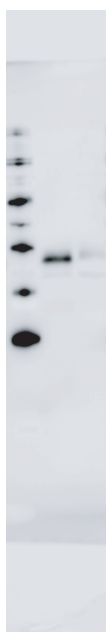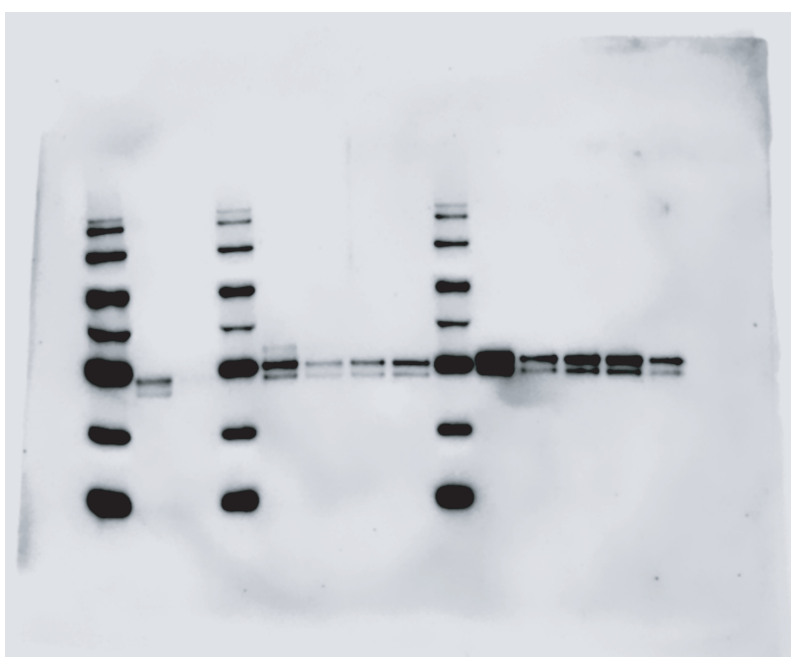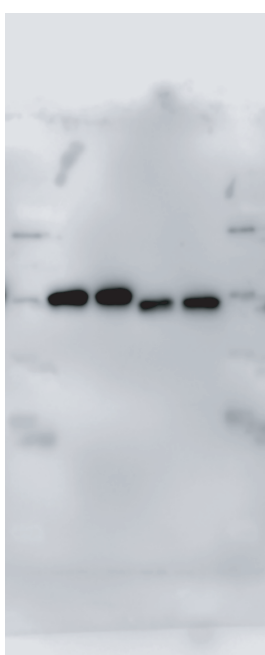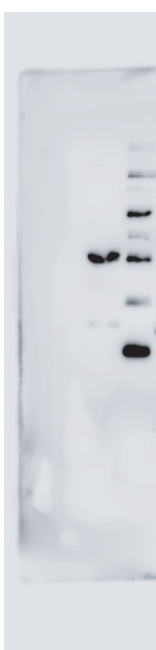

Supplement: Extended Data Fig. 2 — Unprocessed gels and western blots. [file 41557_2022_1004_MOESM11_ESM.pdf]

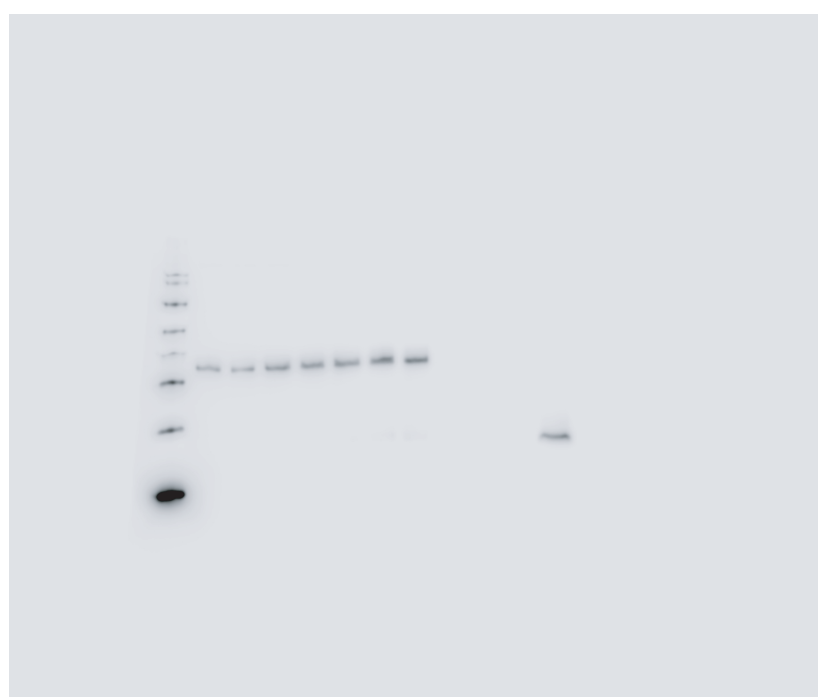

Supplement: Source Data Extended Data Fig. 10 — Unprocessed western blots. [file 41557_2022_1004_MOESM20_ESM.pdf]
